# ﻿First decoding and characterization of the mitogenomes of the crocodile newts *Tylototriton anguliceps* and *T. ngoclinhensis* (Caudata, Salamandridae) from Vietnam and a phylogenetic assessment of the genus *Tylototriton*

**DOI:** 10.3897/zookeys.1265.171020

**Published:** 2025-12-30

**Authors:** Linh Tu Hoang Le, Hoa Thi Ninh, Duy Dinh Vu, Tan Viet Pham, Thomas Ziegler, Tao Thien Nguyen

**Affiliations:** 1 Institute of Biology, Vietnam Academy of Science and Technology, 18 Hoang Quoc Viet Road, Hanoi, Vietnam Institute of Biology, Vietnam Academy of Science and Technology Hanoi Vietnam; 2 Joint Vietnam–Russia Tropical Science and Technology Research Center, 63 Nguyen Van Huyen Road, Hanoi, Vietnam Joint Vietnam–Russia Tropical Science and Technology Research Center Hanoi Vietnam; 3 108 Military Central Hospital, 1 Tran Hung Dao Street, Hanoi, Vietnam 108 Military Central Hospital Hanoi Vietnam; 4 Graduate University of Science and Technology, Vietnam Academy of Science and Technology, 18 Hoang Quoc Viet Road, Hanoi 10072, Vietnam Graduate University of Science and Technology Hanoi Vietnam; 5 Cologne Zoo, Riehler Str. 173, D–50735 Cologne, Germany Cologne Zoo Cologne Germany; 6 Institute of Zoology, University of Cologne, Zülpicher Str. 47b, D-50674 Cologne, Germany University of Cologne Cologne Germany

**Keywords:** 16S rRNA, mitochondrial genome, ND2, phylogenetic analysis, protein-coding genes

## Abstract

*Tylototriton anguliceps* and *T. ngoclinhensis* are two species of crocodile newts native to Vietnam. In this study, we assemble and describe for the first time the complete mitochondrial genomes of *T. anguliceps* (16,720 bp) and *T. ngoclinhensis* (16,260 bp). Both genomes consist of 37 genes, which is consistent with most other vertebrates, including 13 protein-coding genes (PCGs), 22 transfer RNAs (tRNAs), and two ribosomal RNAs (rRNAs). The arrangements of these mitochondrial genes are the same in *T. anguliceps* and *T. ngoclinhensis*, but some genes have slight differences in their lengths, start codons, and stop codons. Phylogenetic analysis using 13 PCGs from the mitogenomes as well as concatenated 16S and ND2 sequences confirm the taxonomic identities of the samples, with *T. anguliceps* belonging to the subgenus Tylototriton and *T. ngoclinhensis* to the subgenus Yaotriton. Furthermore, the phylogenetic analysis reveals that the previously assembled mitogenome (GenBank accession KR733683) thought to represent *T. wenxianensis* is likely belongs to *T. maolanensis*, thus providing the first mitogenome for this species. This study’s re-identification and the completion of two new mitogenomes provide a total of three new mitogenomes for future studies: those of *T. anguliceps*, *T. ngoclinhensis*, and the re-identified *T. maolanensis*. The new mitogenomes can contribute valuable data for future molecular and evolutionary studies, as well as a basis for conservation genomics of the genus *Tylototriton* and other salamanders.

## ﻿Introduction

The genus *Tylototriton* Andersson, 1871, comprising the crocodile newts, inhabits diverse montane forest ecosystems and is distributed across a wide range in South, East, and Southeast Asia, from the eastern Himalayas, eastern Nepal, northern India, Bhutan, Myanmar, central and southern China to Laos, Thailand, and Vietnam (Frost 2025). The complex topography and varied environmental conditions across the distribution of *Tylototriton* have likely contributed to the diversification of this genus. Further surveys in unexplored regions, coupled with the usage of molecular markers and phylogenetic analysis, have contributed to the discovery of numerous new *Tylototriton* species in recent years, such as *T. tongziensis*Li et al., 2022, *T. ngoclinhensis*Phung et al., 2023, *T. soimalai*Pomchote et al., 2024, *T. gaowangjienensis*Huang et al., 2024, and *T. koliaensis*Poyarkov et al., 2024 (Li et al. 2022; Phung et al. 2023; Huang et al. 2024; Pomchote et al. 2024; Poyarkov et al. 2024).

Mitochondrial genomes (mitogenomes) have become indispensable resources in evolutionary biology, offering valuable insights into phylogenetic relationships, population genetics, and molecular evolution, particularly in amphibians (San Mauro et al. 2004). The maternal inheritance, relatively rapid evolutionary rate, and conserved gene content and organization of vertebrate mitogenomes make them highly suitable for inferring evolutionary histories, especially at the species and genus levels (Mueller 2006). Within the genus *Tylototriton*, mitochondrial DNA data have been successfully employed to investigate phylogenetic relationships and delimit species boundaries, revealing cryptic lineages and refining taxonomic classifications (Nishikawa et al. 2013a; Le et al. 2015; Pomchote et al. 2021; Dufresnes and Hernandez 2023). A number of mitochondrial genomes from *Tylototriton* species have been sequenced and assembled; these include *T. wenxianensis* Fei et al., 1984 (Zhang et al. 2008), *T. kweichowensis* Fang & Chang, 1932 (Li et al. 2015) and *T. shanjing* Nussbaum et al., 1995 (Jiang et al. 2015b), *T. ziegleri* Nishikawa et al., 2003 (Jiang et al. 2017), *T. shanorum* Nishikawa et al., 2014 (Zhang et al. 2018b), *T. broadoridgus* Shen et al., 2012, and *T. gaowangjienensis*Huang et al., 2024 (Wang et al. 2022).

This study focuses on two species of *Tylototriton* with distinct distributions and conservation statuses: *Tylototriton anguliceps* Le, Nguyen, Nishikawa, Nguyen, Pham, Matsui, Bernardes & Nguyen, 2015 and *Tylototriton ngoclinhensis* Phung, Pham, Nguyen, Ninh, Nguyen, Bernardes, Le, Ziegler & Nguyen, 2023. *Tylototriton anguliceps*, described in 2015, is recognized by its angular head and is distributed across parts of Thailand, Laos, and Vietnam (Le et al. 2015; Hernandez 2019). It is currently assessed as Least Concern on the IUCN Red List of Threatened Species (IUCN SSC Amphibian Specialist Group 2016) but is considered Endangered according to the Viet Nam Red List of Threatened Species (Pham 2023). *Tylototriton ngoclinhensis* is a recently described species from 2023, endemic to the montane evergreen forests of the Ngoc Linh massif in the Central Highlands of Vietnam and possibly Laos (Phung et al. 2023; WCS 2024). This species inhabits higher elevations compared to many other *Tylototriton* and represents the southernmost known distribution for the genus in Asia. Due to its restricted range and vulnerability to habitat loss, *T. ngoclinhensis* has been proposed for classification as Endangered on the IUCN Red List (Phung et al. 2023).

Given the relatively recent descriptions of these species and the limited availability of comprehensive genomic data, a detailed examination of their mitochondrial genomes is warranted. Complete mitochondrial genome sequences will provide valuable molecular markers for clarifying the phylogenetic placement of *T. anguliceps* and *T. ngoclinhensis* within the genus *Tylototriton*, assessing their genetic divergence from closely related species, and contributing to a better understanding of the evolutionary history and diversification patterns of crocodile newts in Indochina. Furthermore, the analysis of their mitogenomic architecture and characteristics will add to the growing body of data on amphibian mitochondrial genome evolution.

Therefore, for this study we sequenced and characterized the first complete mitochondrial genomes of *T. anguliceps* and *T. ngoclinhensis*. By providing these essential genomic resources, this research will facilitate future phylogenetic and phylogeographic investigations as well as provide essential information for conservation strategies for these species.

## ﻿Methods

### ﻿Sampling and DNA extraction

A sample of *Tylototriton anguliceps* was collected from Xuan Nha Nature Reserve, Son La Province while a sample of *T. ngoclinhensis* was collected from the type locality at Ngoc Linh Mountain, Quang Ngai Province, Central Highlands, Vietnam. The sample specimens were anaesthetized and euthanized in a closed vessel with a piece of cotton wool containing ethyl acetate (Simmons 2002) and then preserved in ethanol. Liver tissue was collected from each sample for molecular analysis and total DNA was extracted.

Qubit 3.0 was used to quantify the DNA concentration and assess for DNA sample quality. The OD_260_/OD_280_ was determined by absorbance measurement while DNA size was determined by agarose gel electrophoresis. Samples meeting the quality criteria of concentration ≥ 2 ng/μL, amount ≥ 90 ng, and OD_260_/OD_280_ ≥ 1.70 were considered suitable for further experimental steps. Samples with DNA size < 1000 bp were flagged.

### ﻿Genome sequencing

After the DNA sample from each species was qualified, it was randomly interrupted using a Covaris ultrasonic crusher, and then the DNA library preparation was completed through end repair, A-tailing, sequencing adapter addition, purification and PCR amplification. After the library was constructed, Qubit v. 3.0 was used for preliminary quantification and library dilution, and then Qsep100 was used to detect the size of the inserted fragment of the library. After the inserted fragment met expectations, the effective concentration of the library was accurately quantified using the Q-PCR method (library effective concentration > 3nM) to ensure the quality of the library.

After the library was qualified, different libraries were pooled to flow cell according to the effective concentration and target data volume. After cBOT clustering, sequencing was performed in paired-end mode using the Illumina high-throughput sequencing platform (HiSeq X) at Bioeditas Technology Corporation (Shaanxi, China; www.bioeditas.cn).

### ﻿Mitochondrial genome assembly and annotation

Based on the short reads from the sequencing platform, the mitochondrial genomes were assembled with MitoZ (Meng et al. 2019). All mitochondrial genomes were also annotated using MitoZ. The obtained GenBank files from MitoZ were used to create visual representations of the mitogenomes using OGDRAW v. 1.3.1 (Greiner et al. 2019). Secondary structures of transfer RNA (tRNA) were predicted using tRNAscan-SE v. 2.0 (Chan et al. 2021). The Relative Synonymous Codon Usage (RSCU) values of both whole mitogenomes were computed using MEGA 11 (Tamura et al. 2021).

### ﻿Phylogenetic tree construction

To evaluate the phylogenetic relationships of the two newly sequenced species in this study, mitochondrial genomes from another 13 *Tylototriton* species and four other outgroup species in the Salamandridae family were downloaded from GenBank, NCBI (Suppl. material 1: table S1). All of the 13 Protein Coding Genes (PCGs) were extracted and then individually aligned based on the 19 studied species in MEGA 11 using the MUSCLE algorithm (Edgar 2004; Tamura et al. 2021). Afterwards, the PCG alignments were concatenated to make a combined dataset and ModelFinder was used to find the best-fit model for each partition (Kalyaanamoorthy et al. 2017). Phylogenetic trees were constructed via both Bayesian-inference (BI) and maximum-likelihood (ML) methods. BI trees were constructed with MrBayes v. 3.2.7 running 10,000,000 generations and sampling every 100 generations; after discarding the first 25% samples as burn-in, posterior probabilities (PP) were calculated into a consensus tree (Ronquist et al. 2012). Meanwhile, ML trees were made using IQ-TREE v. 2.3.5 (Bui et al. 2020) with 10,000 ultrafast bootstrap replicates. In addition to phylogenetic analysis using the 13 PCGs from known mitochondrial genomes, a phylogenetic tree was also created using concatenated sequences of 16S rRNA and ND2 gene to confirm the identities of the examined mitochondrial genomes. Suppl. material 1: table S2 lists the 16S rRNA and ND2 sequences used in the phylogenetic analysis.

## ﻿Results

### ﻿Mitogenome assembly and organization

**Figure 1.**
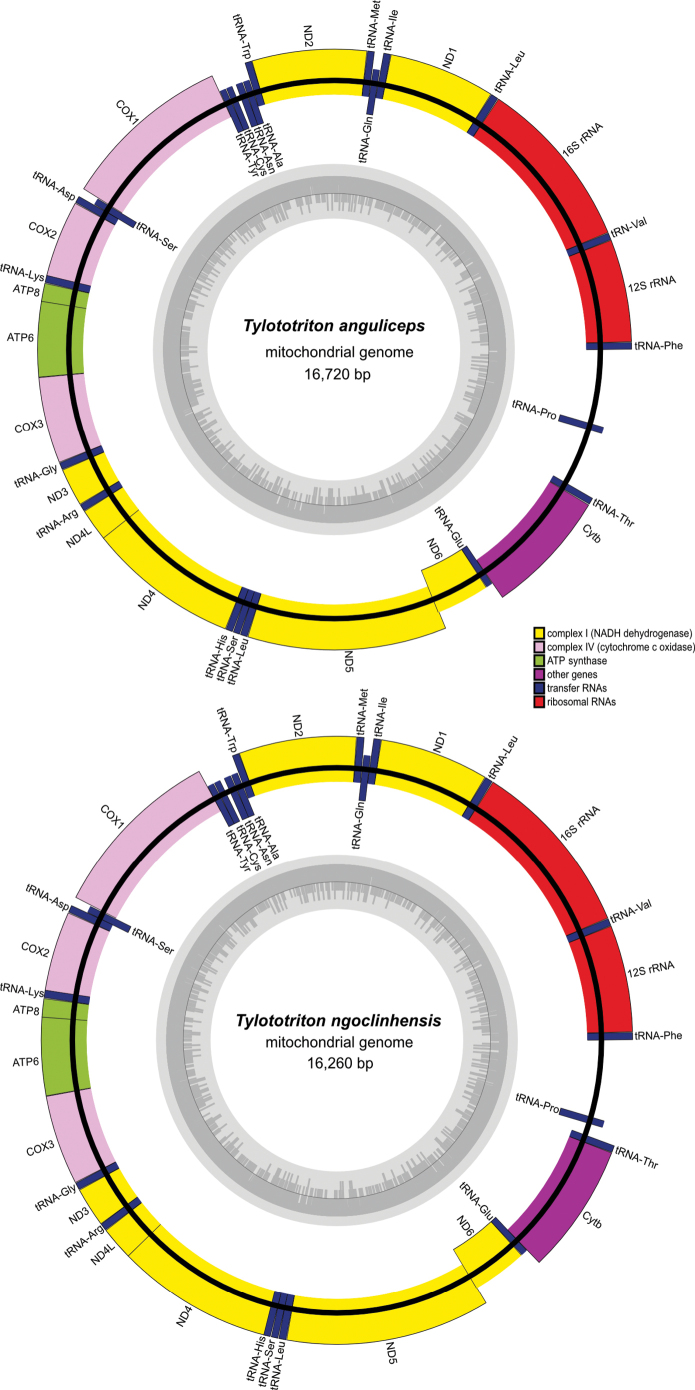
Gene maps of the mitochondrial genomes of *Tylototriton anguliceps* and *T. ngoclinhensis*.

Sequencing and assembly statistics can be seen in Table 1. The assembled circular mitogenomes of *Tylototriton anguliceps* and *T. ngoclinhensis* (GenBank accession numbers: PX673615 and PX673616) were 16,720 bp and 16,260 bp, respectively. Annotations provided by MitoZ showed that both mitogenomes are composed of 37 genes typically found in salamander mitogenomes including 2 rRNA genes (12S rRNA and 16S rRNA), 13 PCGs (ND1, ND2, COX1, COX2, ATP8, ATP6, COX3, ND3, ND4L, ND4, ND5, ND6 and Cytb), 22 tRNAs, and a control region (Fig. 1, Table 2). A noncoding control region, also known as D-loop, is located between tRNA-Phe and tRNA-Pro. The gene organizations for both mitogenomes are also identical, with most genes located on the (+) strand, with the exception of ND6 and eight tRNA genes (tRNA-Pro, tRNA-Glu, tRNA-Ser, tRNA-Tyr, tRNA-Cys, tRNA-Asn, tRNA-Ala, and tRNA-Gln) residing on the (-) strand. The mitogenome of *T. anguliceps* is slightly longer than that of *T. ngoclinhensis* partly due to an expansion between tRNA-Pro and tRNA-Thr (Fig. 1).

**Table 1.** Assembly statistics of two samples from *Tylototriton anguliceps* and *T. ngoclinhensis*.

Item	*T. anguliceps*	*T. ngoclinhensis*
Voucher ID	TY000001	TY000002
Assemble data set(G)	10	10
Assemble reads pairs	14029	7714
Mean coverage	251X	142X

**Table 2.** Mitochondrial genome organization of *Tylototriton anguliceps* and *T. ngoclinhensis* (IN: intergenic nucleotides).

Gene name	*T. anguliceps*						*T. ngoclinhensis*						Anticodon	Strand
	Start	End	Length (bp)	IN	Start Codon	Stop Codon	Start	End	Length (bp)	IN	Start Codon	Stop Codon		
tRNA-Phe	1	69	69	0			1	70	70	0			GAA	+
12S rRNA	70	996	927	-1			71	999	929	-1				+
tRNA-Val	996	1065	70	0			999	1068	70	0			TAC	+
16S rRNA	1066	2627	1562	2			1069	2631	1563	2				+
tRNA-Leu1	2629	2703	75	0			2633	2707	75	0			TAA	+
ND1	2704	3672	969	-1	ATG	TAG	2708	3676	969	-1	ATG	TAG		+
tRNA-Ile	3672	3742	71	3			3676	3746	71	2			GAT	+
tRNA-Gln	3746	3815	70	2			3749	3819	71	2			TTG	-
tRNA-Met	3818	3887	70	0			3822	3891	70	0			CAT	+
ND2	3888	4931	1044	-2	ATG	TAA	3892	4935	1044	-2	ATG	TAA		+
tRNA-Trp	4930	4998	69	1			4934	5002	69	1			TCA	+
tRNA-Ala	5000	5068	69	0			5004	5072	69	0			TGC	-
tRNA-Asn	5069	5141	73	32			5073	5145	73	34			GTT	-
tRNA-Cys	5174	5239	66	0			5180	5245	66	0			GCA	-
tRNA-Tyr	5240	5306	67	1			5246	5313	68	1			GTA	-
COX1	5308	6858	1551	0	GTG	TAA	5315	6865	1551	0	GTG	TAA		+
tRNA-Ser	6859	6929	71	1			6866	6936	71	1			TGA	-
tRNA-Asp	6931	7000	70	1			6938	7007	70	1			GTC	+
COX2	7002	7689	688	0	ATG	T(AA)	7009	7696	688	0	ATG	T(AA)		+
tRNA-Lys	7690	7762	73	1			7697	7769	73	1			TTT	+
ATP8	7764	7931	168	-10	ATG	TAA	7771	7935	165	-10	ATG	TAA		+
ATP6	7922	8605	684	-1	ATG	TAA	7926	8609	684	-1	ATG	TAA		+
COX3	8605	9388	784	0	ATG	T(AA)	8609	9392	784	0	ATG	T(AA)		+
tRNA-Gly	9389	9458	70	0			9393	9461	69	0			TCC	+
ND3	9459	9804	346	0	ATG	T(AA)	9462	9809	348	-2	ATG	TAA		+
tRNA-Arg	9805	9873	69	0			9808	9876	69	0			TCG	+
ND4L	9874	10170	297	-7	ATG	TAA	9877	10173	297	-7	ATG	TAA		+
ND4	10164	11541	1378	0	ATG	T(AA)	10167	11544	1378	0	ATG	T(AA)		+
tRNA-His	11542	11610	69	0			11545	11612	68	0			GTG	+
tRNA-Ser	11611	11678	68	-1			11613	11680	68	-1			GCT	+
tRNA-Leu2	11678	11749	72	0			11680	11751	72	0			TAG	+
ND5	11750	13561	1812	-15	ATG	TAA	11752	13563	1812	-15	ATG	TAA		+
ND6	13547	14065	519	0	ATG	AGA	13549	14067	519	0	ATG	AGA		-
tRNA-Glu	14066	14133	68	1			14068	14135	68	2			TTC	-
Cytb	14135	15272	1138	3	ATG	T(AA)	14138	15279	1142	-1	ATG	T(AA)		+
tRNA-Thr	15276	15343	68	569			15279	15346	68	108			TGT	+
tRNA-Pro	15913	15984	72	0			15455	15525	71	0			TGG	-
D-loop	15985	16720	736	0			15526	16260	735	0				+

### ﻿Protein-coding genes and codon usage

As seen in Table 3, both mitogenomes display fairly similar base compositions: *T. anguliceps*: A (33.6%), T (25.0%), G (14.6%), and C (26.8%); *T. ngoclinhensis*: A (33.8%), T (25.6%), G (14.5%), and C (26.1%). Similar to the nucleotide compositions of other *Tylototriton* species (Wang et al. 2022), both *T. anguliceps* and *T. ngoclinhensis* have nucleotide composition patterns that show high A + T contents (58.6% and 59.4%), positive AT-skewness (0.15 and 0.14) and negative GC-skewness (both -0.29) (Table 3).

**Table 3.** Nucleotide composition and skewness of the mitogenomes of *T. anguliceps* and *T. ngoclinhensis*; the values for *T. anguliceps* are shown before the slash (/) and of *T. ngoclinhensis* are listed after the slash.

Region	T (%)	C (%)	A (%)	G (%)	A+T (%)	AT-skew	GC-skew
ATP6	25.3 / 27.2	30.7 / 29.8	31.7 / 30.8	12.3 / 12.1	57 / 58	0.11 / 0.06	-0.43 / -0.42
ATP8	23.8 / 24.2	26.8 / 26.1	42.3 / 40.0	7.1 / 9.7	66.1 / 64.2	0.28 / 0.25	-0.58 / -0.46
COX1	27.9 / 28.7	27.3 / 26.6	26.4 / 27.3	18.3 / 17.3	54.3 / 56	-0.03 / -0.03	-0.20 / -0.21
COX2	25.0 / 25.1	26.0 / 25.8	33.9 / 34.0	15.1 / 15.1	58.9 / 59.1	0.15 / 0.15	-0.26 / -0.26
COX3	25.1 / 27.3	29.8 / 27.6	27.8 / 28.1	17.2 / 17.1	52.9 / 55.4	0.05 / 0.01	-0.27 / -0.23
Cytb	25.7 / 25.8	29.3 / 29.0	30.3 / 29.9	14.6 / 15.2	56 / 55.7	0.08 / 0.07	-0.33 / -0.31
ND1	27.1 / 26.6	25.6 / 26.2	32.5 / 33.5	14.8 / 13.6	59.6 / 60.1	0.09 / 0.11	-0.27 / -0.32
ND2	23.7 / 23.3	28.4 / 29.1	37.1 / 38.0	10.9 / 9.6	60.8 / 61.3	0.22 / 0.24	-0.45 / -0.50
ND3	26.9 / 29.0	30.6 / 27.0	27.5 / 29.3	15.0 / 14.7	54.4 / 58.3	0.01 / 0.01	-0.34 / -0.29
ND4	24.8 / 23.9	29.9 / 30.6	32.7 / 32.3	12.6 / 13.2	57.5 / 56.7	0.14 / 0.15	-0.41 / -0.39
ND4L	27.9 / 27.6	27.3 / 27.6	32.0 / 31.6	12.8 / 13.1	59.9 / 59.2	0.07 / 0.07	-0.36 / -0.36
ND5	26.6 / 27.2	27.0 / 25.7	34.3 / 34.8	12.1 / 12.3	60.9 / 62	0.13 / 0.12	-0.38 / -0.35
ND6	46.1 / 45.3	10.2 / 11.9	11.6 / 12.1	32.2 / 30.6	57.7 / 57.4	-0.60 / -0.58	0.52 / 0.44
PCGs	25.3 / 25.7	28.3 / 27.8	32.5 / 32.7	13.9 / 13.8	57.8 / 58.4	0.12 / 0.12	-0.34 / -0.34
tRNAs	26.8 / 26.6	22.9 / 23.0	34.7 / 34.5	15.6 / 15.9	61.5 / 61.1	0.13 / 0.13	-0.19 / -0.18
D-loop	34.8 / 35.6	21.7 / 20.1	27.6 / 29.0	15.9 / 15.2	62.4 / 64.6	-0.12 / -0.10	-0.15 / -0.14
12S rRNA	19.8 / 19.7	23.8 / 24.0	37.6 / 38.3	18.7 / 18.0	57.4 / 58	0.31 / 0.32	-0.12 / -0.14
16S rRNA	23.2 / 23.4	20.0 / 19.8	40.9 / 40.9	15.8 / 16.0	64.1 / 64.3	0.28 / 0.27	-0.12 / -0.11
Genome	25.0 / 25.6	26.8 / 26.1	33.6 / 33.8	14.6 / 14.5	58.6 / 59.3	0.15 / 0.14	-0.29 / -0.29

The majority of PCGs found in *T. anguliceps* and *T. ngoclinhensis* have start codons of ATG except for the COX1 gene, which starts with GTG (Table 1). Among the four kinds of stop codons used by the PCGs, TAA is used by most PCGs (ND2, COX1, ATP8, ATP6, ND4L, and ND5) in both species but the ND3 gene of *T. anguliceps* ends with T(AA) instead of TAA like in *T. ngoclinhensis*. ND1 gene ends with TAG and ND6 ends with AGA, while the remaining genes COX2, COX3, ND3, ND4, and Cytb end with the incomplete stop codon T(AA).

The 13 PCGs found in *T. anguliceps* and *T. ngoclinhensis* constitute a total length of 11,378 bp and 11,381 bp, respectively (Table 3). The base composition of PCGs found in *T. anguliceps* is A (32.5%), T (25.3%), G (13.9%), and C (28.3%), while in *T. ngoclinhensis* is A (32.7%), T (25.7%), G (13.8%), and C (27.8%). From these base compositions, the A + T contents of the PCGs in *T. anguliceps* is calculated to be 57.8% and 58.4% for *T. ngoclinhensis*, corresponding to positive AT-skew of 0.12 and negative GC-skew of -0.34 for both species. In both species, the majority of PCGs show positive AT-skew apart from two genes, COX1 and ND6, which have negative values, while all PCGs have negative GC-skew except for ND6.

**Figure 2.**
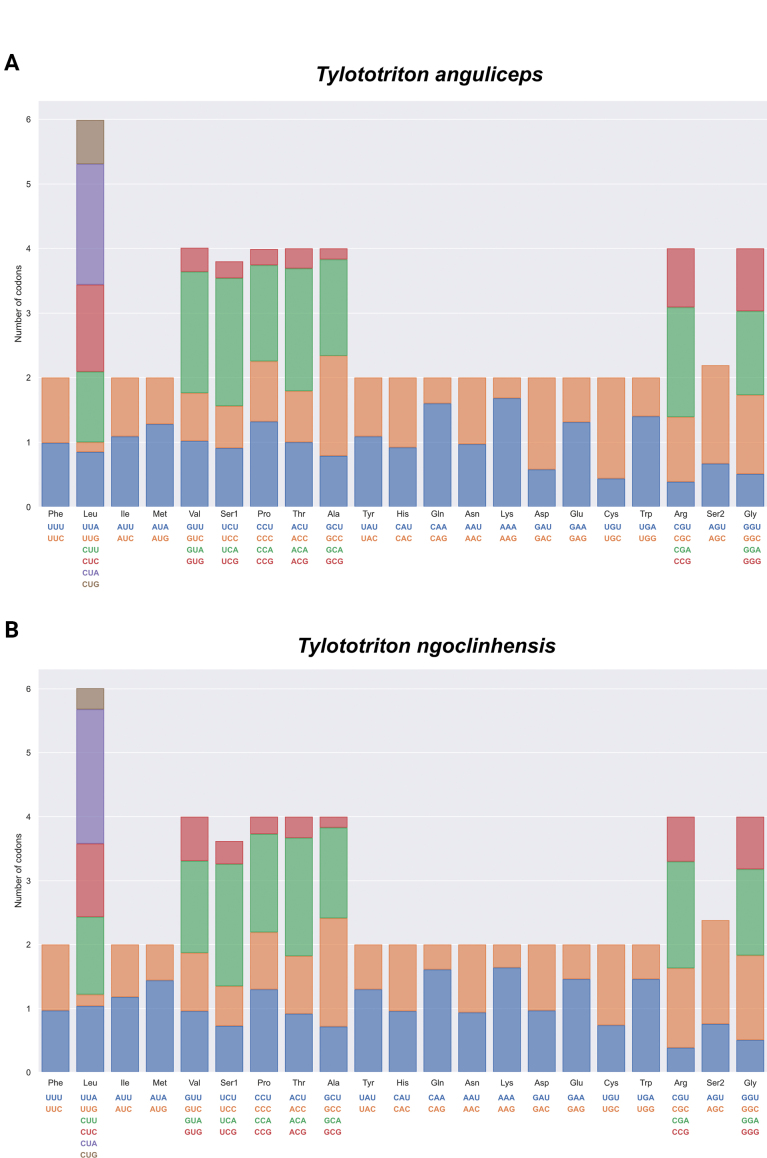
Relative Synonymous Codon Usage (RSCU) of the PCGs in mitogenomes. **A.***Tylototriton anguliceps*; **B.***T. ngoclinhensis*.

The relative synonymous codon usage (RSCU) of *T. anguliceps* and *T. ngoclinhensis* are summarized in Fig. 2. Codons with RSCU values > 1.0 have positive codon usage bias (CUB) and are defined as abundant codons, whereas those with RSCU values < 1.0 have negative CUB and are defined as less-abundant codons (Gun et al. 2018). Overall, RSCU from both species are relatively similar to each other but GUA (Val) seems to be more abundant in *T. anguliceps* than in *T. ngoclinhensis*. In terms of codon frequencies, CUA (Leu), GUA (Ser1), ACA (Thr), CAA (Gln), and AAA (Lys) appear to be the most abundant codons whereas UUG (Leu) and GCG (Ala) have the lowest codon frequencies in both studied species.

### ﻿Ribosomal RNAs, transfer RNA, and control region

Two typical types of ribosomal units are found in the mitochondrial genomes of *T. anguliceps* and *T. ngoclinhensis*: the small 12S rRNA and the large 16S rRNA subunits. The 12S rRNA gene is located between tRNA-Phe and tRNA-Val with lengths of approximately 927–929 bp. The 16S rRNA is positioned between tRNA-Val and tRNA-Leu1, ranging from 1562 to 1563 bp in length.

**Figure 3.**
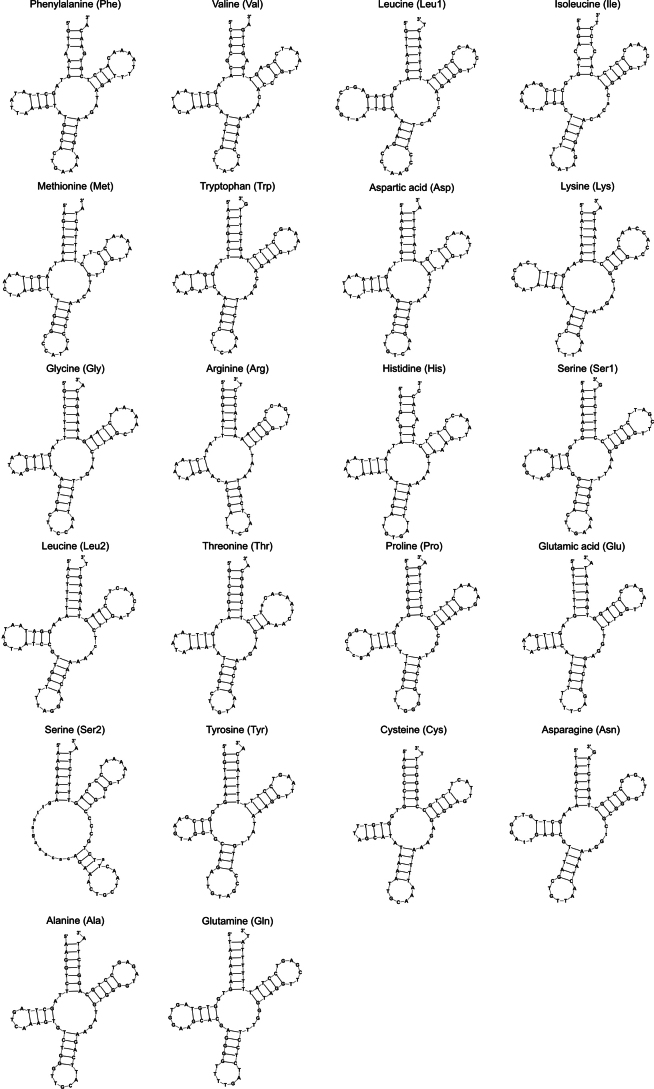
Secondary structure of the 22 tRNA genes of the mitochondrial genome of *Tylototriton anguliceps* predicted by tRNAScan-SE 2.0.

**Figure 4.**
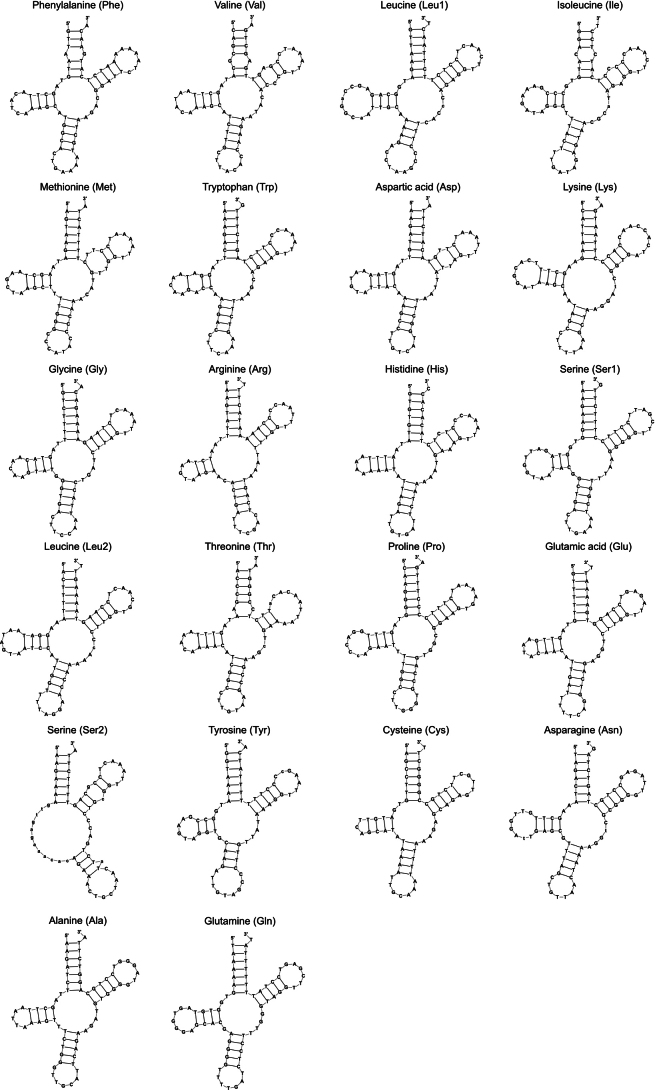
Secondary structure of the 22 tRNA genes of the mitochondrial genome of *Tylototriton ngoclinhensis* predicted by tRNAScan-SE 2.0.

*Tylototriton anguliceps* and *T. ngoclinhensis* both have 22 tRNA genes (eight on the L-strand and 14 on the H-strand). Their individual gene lengths ranged from 66 bp (tRNA-Cys) to 75 bp (tRNA-Leu1) with most tRNA genes having an identical length for both species except for the following genes: tRNA-Phe, tRNA-Gln, tRNA-Tyr, tRNA-Gly, tRNA-His, and tRNA-Pro (Table 2). All tRNA genes, except for tRNA-Ser2, can be folded into a typical cloverleaf structure as predicted by tRNAScan-SE 2.0 (Figs 3, 4). In both species and other *Tylototriton* species, compared to tRNA-Ser1, tRNA-Ser2 is missing the D-arm, and the T-arm has a mismatch near the middle of the stem. tRNA-Trp also has a mismatch in the stem of the D-arm. Majority of tRNAs have 5 bp in the anticodon stem except for those with only 4 bp, like tRNA-Leu1, tRNA-Lys, tRNA-Ser2, and tRNA-Tyr.

The Control Region (CR), also known as the D-loop, represents the primary non-coding portion of the mitochondrial genome in vertebrates. It is crucial for initiating and regulating mitochondrial gene replication and transcription. In *T. anguliceps* and *T. ngoclinhensis*, the CR is located between tRNA-Pro and tRNA-Phe with a length of 736 bp and 735 bp, respectively. The obtained lengths are slightly longer than the control regions found in other *Tylototriton* species such as *T. broadoridgus* (716 bp) and *T. gaowangjienensis* (715 bp) (Wang et al. 2022).

### ﻿Phylogenetic analysis

Phylogenetic analysis was performed using 13 PCGs from 16 *Tylototriton* mitogenomes along with five mitochondrial genomes of four species as outgroups. The aligned and concatenated PCGs from the 21 mitogenomes were tested for best substitution model in ML analysis for each partition using ModelFinder. The models were then translated to the closest approximate model implemented in MrBayes for BI analysis. The results of substitution model selection for ML and BI analyses can be seen in Table 4. There was a greater variety of models chosen for ML analysis compared to BI analysis due to IQ-TREE2 implementing more substitution models. Furthermore, the most common model chosen for BI analysis is the GTR+I+G4, one of the most preferred models used in molecular phylogenetic analysis (Barba-Montoya et al. 2020).

**Table 4.** Best partitioning models chosen for maximum-likelihood (ML) and Bayesian-inference (BI) analyses using PCGs.

Gene	Best model for ML analysis	Best model for BI analysis
ATP6	TIM3+F+I+G4	GTR+I+G4
ATP8	TIM3+F+G4	GTR+G4
COX1	TIM3+F+I+G4	GTR+I+G4
COX2	TN+F+G4	GTR+G4
COX3	TIM3+F+I+G4	GTR+I+G4
Cytb	TN+F+R3	GTR+G3
ND1	TIM2+F+G4	GTR+G4
ND2	TIM+F+I+G4	GTR+I+G4
ND3	K3Pu+F+G4	GTR+G4
ND4L	TIM+F+I	GTR+I
ND4	HKY+F+I+G4	HKY+I+G4
ND5	TIM2+F+I+G4	GTR+I+G4
ND6	TN+F+G4	GTR+G4

**Figure 5.**
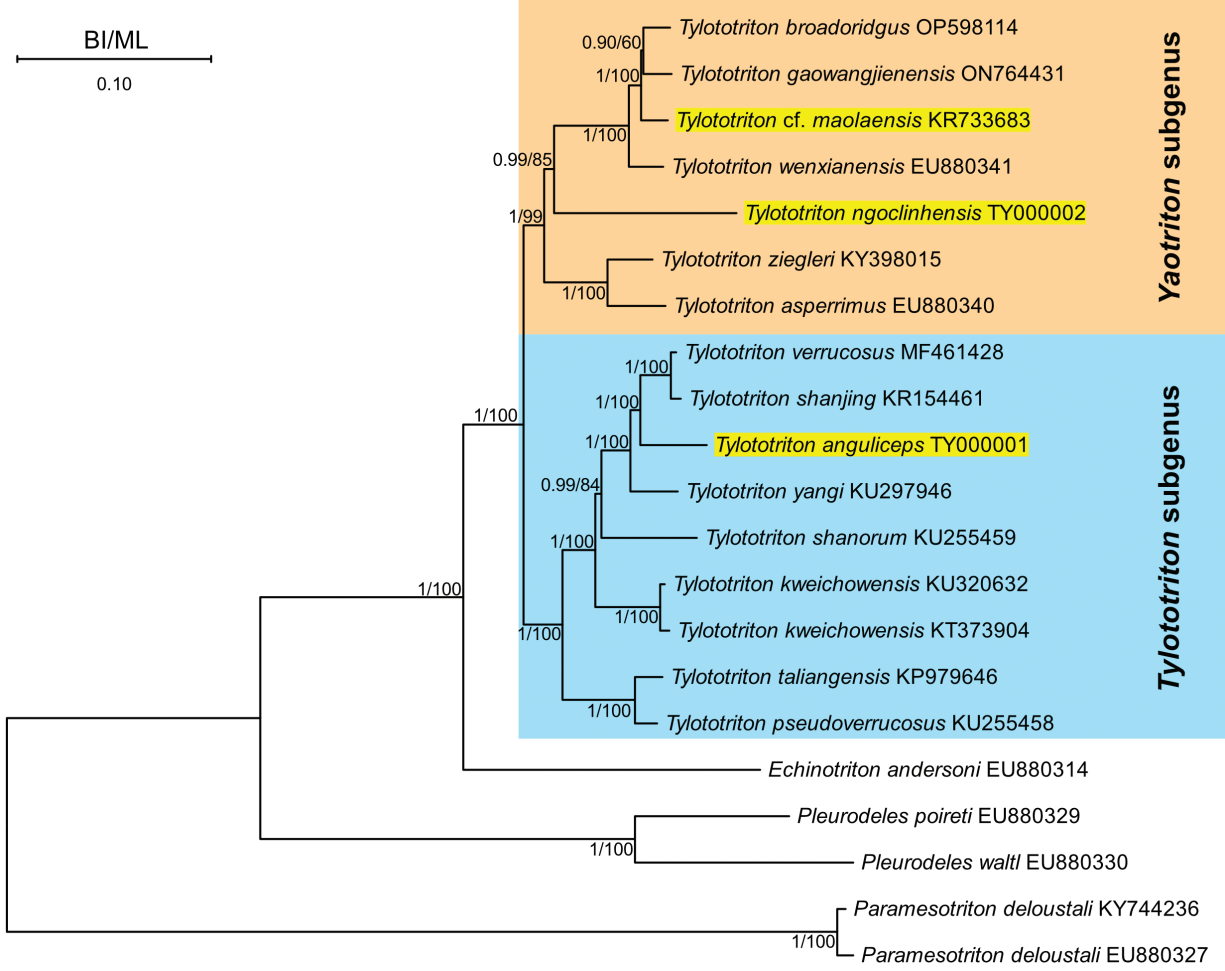
Phylogenetic tree based on 13 PCGs found in known *Tylototriton* mitochondrial genomes using BI and ML analysis. Values at each node indicate Bayesian Posterior Probabilities and Ultrafast Bootstrap Values respectively (BI/ML).

Our phylogenetic analysis based on the 13 PCGs corroborates the “Primitive newts” clade identified by Wang et al. (2022) (Fig. 5). The genus *Tylototriton* consistently formed a strongly supported monophyletic group (PP/UFB = 1/100), further subdividing into two well-supported subgenera: *Yaotriton* and *Tylototriton*. Within the subgenus Yaotriton, *T. ngoclinhensis* and *T. vietnamensis* are identified as the earliest diverging branches, while *T. anguliceps* is nested within a clade sharing a common ancestor with *T. verrucosus* and *T. shanjing*. Notably, two individuals, KR733683 (Han et al. 2016) and EU880341 (Zhang et al. 2008), were both previously classified as *T. wenxianensis*, but our analysis shows that they are not monophyletic but are rather paraphyletic to each other. Thus, further analysis using 16S rRNA and ND2 genes is needed to elucidate the correct taxonomic status of these two samples.

**Figure 6.**
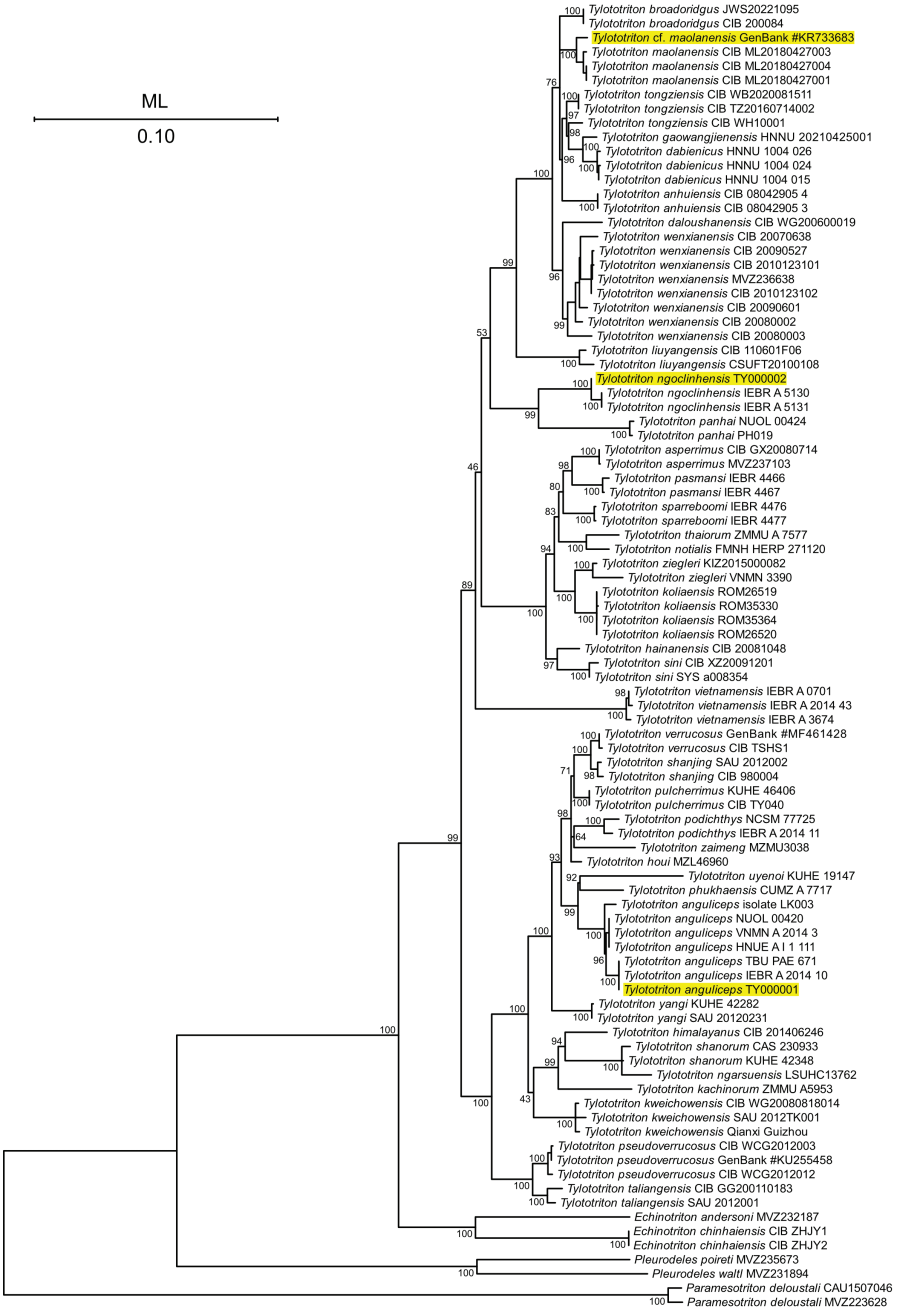
Phylogenetic tree based on concatenated 16S and ND2 sequences of known *Tylototriton* species using ML analysis. Ultrafast Bootstrap Value is shown at each node. Voucher numbers are listed after species names. In cases where the voucher is unknown, GenBank accession number is provided.

For the phylogenetic analysis utilizing concatenated 16S rRNA and ND2 sequences, the optimal models selected for ML analysis were TIM2+F+I+R2 for the 16S gene and TIM+F+R3 for the ND2 gene. Generally, the phylogenetic relationships inferred from the concatenated 16S rRNA and ND2 sequences demonstrate congruence with those obtained from the PCG analysis (Fig. 6). For instance, TY000001 clusters with other members of *T. anguliceps*, forming a sister relationship with *T. phukhaensis* and *T. uyenoi*. *Tylototriton ngoclinhensis* (TY000002) is also placed in the same clade as other *T. ngoclinhensis* based on the ND2 sequences of two other *T. ngoclinhensis* samples (voucher number IEBR A.5130 and IEBR A.5131) (Fig. 6).

As previously mentioned, phylogenetic analysis of 13 PCGs suggests that the specimen KR733683 may have been misidentified as *T. wenxianensis*. Our analysis, utilizing known 16S and ND2 sequences, places KR733683 within the same clade as *T. maolanensis*, not *T. wenxianensis*. This finding is further supported by the specimen’s collection location in Libo County, Guizhou Province, China—the same county where *T. maolanensis* was later described (Li et al. 2020). Therefore, evidence indicates that KR733683 is a member of *T. maolanensis*, contrary to its original description by Han et al. (2016). Furthermore, as no mitochondrial genome for *T. maolanensis* has been published to date, KR733683 likely represents the first sequenced mitochondrial genome for this species.

## ﻿Discussion

The present study provides the first complete mitochondrial genome sequences for *Tylototriton anguliceps* and *T. ngoclinhensis* collected from Vietnam. Both species exhibit the typical vertebrate mitochondrial gene arrangement, comprising 13 PCGs, 22 tRNAs, 2 rRNAs, and a control region (D-loop). The newly assembled mitogenomes exhibit strong similarities to each other and to other known *Tylototriton* mitogenomes regarding size, organization, nucleotide composition of various regions, and codon usage of PCGs. Despite these general similarities, minor differences were also observed. For instance, the ND3 gene of *T. anguliceps* terminates with T(AA) and that of *T. taliangensis* ends with TAG, contrasting with the TAA stop codon found in *T. ngoclinhensis*, *T. broadoridgus*, and *T. gaowangjienensis*. Additionally, subtle variations in the lengths and positions of certain genes and control regions were noted. The functional or evolutionary significance of these genomic differences remains unclear, warranting further investigation in future studies.

Our phylogenetic analysis of the genus *Tylototriton* utilized all available mitogenomes from GenBank, supplemented by two newly sequenced genomes from this study. This dataset, covering 15 of the 43 recognized species (Frost 2025), produced a phylogeny largely consistent with previous research showing a robust split between two principal clades corresponding to the subgenera *Yaotriton* and *Tylototriton* (Han et al. 2016; Wang et al. 2022; Dufresnes and Hernandez 2023). Notably, we re-identified one mitogenome (GenBank: KR733683) as *T. maolanensis*, contrary to its original designation as *T. wenxianensis*. This reclassification is justified by two key points: the specimen originates from the type locality of *T. maolanensis*, and it forms a clade with other *T. maolanensis* sequences with strong support in our phylogenetic tree. This revision highlights the critical need for continuous taxonomic and phylogenetic research as well as sound reference specimen selection and identification to maintain accurate genetic resources, which are foundational for future studies and effective conservation efforts.

Beyond methodological considerations, our newly generated mitogenomes hold direct conservation value. *Tylototriton anguliceps* and *T. ngoclinhensis* occur in geographically restricted highland habitats of Vietnam, environments that are particularly vulnerable to anthropogenic threats such as habitat destruction, agricultural expansion, and climate change. Thus, monitoring via genetic markers can provide valuable information for conservation efforts of these vulnerable species. Complete mitochondrial sequences provide a critical foundation for designing molecular markers tailored to biodiversity monitoring. In particular, the data generated here can be used to develop species-specific primers and probes for environmental DNA (eDNA) assays, an increasingly powerful non-invasive tool for detecting rare and elusive amphibians (Diver et al. 2024). In the genus *Tylototriton*, only a single eDNA-based detection study exists for *T. uyenoi* (Osathanunkul and Minamoto 2021). Targeting short, highly specific mitochondrial fragments (e.g. within ND2 or COX1) could facilitate qPCR or ddPCR assays for *T. anguliceps* and *T. ngoclinhensis*. Thus, the assembled mitogenomes from *T. anguliceps* and *T. ngoclinhensis* can provide crucial resources for future eDNA and other conservation genetics research.

Despite the novel contributions of this work, several limitations must be acknowledged. First, our data rely on a single individual per species (*T. anguliceps* and *T. ngoclinhensis*). This limited sampling precludes assessment of intra-specific diversity, heteroplasmy, and possible geographic structuring of mitochondrial haplotypes. Consequently, the present results should be regarded as representative but preliminary, and expanded sampling across populations will be essential to capture the full spectrum of mitochondrial diversity not just at the genus level but also at the species level. Second, reliance exclusively on short-read Illumina data raises the possibility of nuclear mitochondrial pseudogenes being incorporated into assemblies (Bensasson et al. 2001). Although no frame-shifts or premature stop codons were detected in the PCGs reported here, further validation through read-mapping coverage analysis or PCR-Sanger sequencing of repetitive and control regions is recommended to rule out assembly artifacts.

This study represents a foundational step in elucidating mitogenomic characteristics and phylogenetic relationships of *Tylototriton* in Vietnam. Future research should expand geographic sampling, integrate nuclear genomic data, and investigate gene-wise selection patterns (e.g. dN/dS ratios) to identify signals of adaptive evolution. Validation of repetitive regions through PCR-Sanger sequencing, coupled with divergence-time estimation and biogeographic analyses, will further strengthen understanding of lineage history. Integrating these molecular approaches with ecological and morphological datasets will be crucial for reconstructing the evolutionary history of *Tylototriton* and for informing conservation strategies targeting these rare and threatened amphibians in Vietnam.

## ﻿Conclusion

We report the first mitochondrial genomes and their characteristics for *Tylototriton anguliceps* and *T. ngoclinhensis*. The gene structure, RNA secondary structure, base composition and phylogenetic relationships were analysed. The results contribute to the mitochondrial genome knowledge of *Tylototriton* and provide molecular and genetic information for species conservation, molecular identification and *Tylototriton* species evolution.

## ﻿Additional information

### Use of AI

No use of AI was reported.

### Author ORCIDs

Linh Tu Hoang Le https://orcid.org/0009-0003-0887-2786

Hoa Thi Ninh https://orcid.org/0009-0002-8133-432X

Duy Dinh Vu https://orcid.org/0000-0001-5677-6965

Tan Viet Pham https://orcid.org/0009-0008-7564-9409

Thomas Ziegler https://orcid.org/0000-0002-4797-609X

Tao Thien Nguyen https://orcid.org/0000-0002-5640-4536

### Data availability

The assembled mitogenome sequences have been deposited in the GenBank database, NCBI (https://www.ncbi.nlm.nih.gov/) under accession numbers: PX673615 (*T. anguliceps*) and PX673616 (*T. ngoclinhensis*). All data that support the findings of this study are available in the main text, Supplementary materials, or from the corresponding authors upon reasonable request.

## Supplementary materials

Supplementary material 1 Sequences used in phylogenetic analysis Data type xlsx Explanation note Mitochondrial genomes of 13 *Tylototriton* species and four other outgroup species in the Salamandridae family, downloaded from GenBank, NCBI, and 16S rRNA and ND2 sequences used in the phylogenetic analysis. https://binary.pensoft.net/file/1502671This dataset is made available under the Open Database License (http://opendatacommons.org/licenses/odbl/1.0/). The Open Database License (ODbL) is a license agreement intended to allow users to freely share, modify, and use this Dataset while maintaining this same freedom for others, provided that the original source and author(s) are credited.Linh Tu Hoang Le, Hoa Thi Ninh, Duy Dinh Vu, Tan Viet Pham, Thomas Ziegler, Tao Thien Nguyen

Supplementary material 2 References for sequences used in phylogenetic analysis Data type docx https://binary.pensoft.net/file/1502672This dataset is made available under the Open Database License (http://opendatacommons.org/licenses/odbl/1.0/). The Open Database License (ODbL) is a license agreement intended to allow users to freely share, modify, and use this Dataset while maintaining this same freedom for others, provided that the original source and author(s) are credited.Linh Tu Hoang Le, Hoa Thi Ninh, Duy Dinh Vu, Tan Viet Pham, Thomas Ziegler, Tao Thien Nguyen
