# Supplementary material for: ﻿First decoding and characterization of the mitogenomes of the crocodile newts Tylototriton anguliceps and T. ngoclinhensis (Caudata, Salamandridae) from Vietnam and a phylogenetic assessment of the genus Tylototriton
Source: Zookeys. 2025 Dec 30;1265:129–50. doi: 10.3897/zookeys.1265.171020 (PMC12776023; doi:10.3897/zookeys.1265.171020)
Supplement: Supplementary material 2 — References for sequences used in phylogenetic analysis [file zookeys-1265-129_article-171020__-s002.docx]

**File S3. References for sequences used in phylogenetic analysis:**

Bernardes M, Pham TC, Nguyen QT, Le MD, Bonkowski M, Ziegler T (2017) Comparative morphometrics and ecology of a newly discovered population of *Tylototriton vietnamensis* from northeastern Vietnam including remarks on species conservation. Salamandra (Frankfurt) 53(3): 451–457.

Bernardes M, Le MD, Nguyen TQ, Pham CT, Pham AV, Nguyen TT, Rödder D, Bonkowski M, Ziegler T (2020) Integrative taxonomy reveals three new taxa within the *Tylototriton asperrimus* complex (Caudata, Salamandridae) from Vietnam. ZooKeys 935: 121–164. <https://doi.org/10.3897/zookeys.935.37138>

Decemson H, Lalremsanga HT, Elangbam PS, Vabeiryureilai M, Shinde P, Purkayastha J, Arkhipov DV, Bragin AM, Poyarkov NA (2023) A new cryptic species of *Tylototriton* (Amphibia, Caudata, Salamandridae) from mysterious mountain lakes in Manipur, north-eastern India. Herpetozoa (Wien) 36: 203–224. <https://doi.org/10.3897/herpetozoa.36.e106614>

Dufresnes C, Hernandez A (2023) Towards completing the crocodile newts’ puzzle with all-inclusive phylogeographic resources. Zoological Journal of the Linnean Society 197(3): 620–640. <https://doi.org/10.1093/zoolinnean/zlac038>

Grismer LL, Wood PLJ, Quah ESH, Thura MK, Espinoza RE, Grismer MS, Murdoch ML, Lin A (2018) A new species of crocodile newt *Tylototriton* (Caudata: Salamandridae) from Shan State, Myanmar (Burma). Zootaxa 4500(4): 553–573. <https://doi.org/10.11646/zootaxa.4500.4.5>

Grismer LL, Wood PL, Quah ESH, Thura MK, Espinoza RE, Murdoch ML (2019) A new species of crocodile newt *Tylototriton* (Caudata: Salamandridae) from northern Myanmar (Burma). Journal of Natural History 53(7–8): 475–495. <https://doi.org/10.1080/00222933.2019.1587534>

Han F, Jiang Y, Zhang M (2016) The complete mitochondrial genome sequence of Wenxian knobby newt *Tylototriton wenxianensis* (Amphibia: Caudata). Mitochondrial DNA. Part A, DNA Mapping, Sequencing, and Analysis 27(4): 2901–2902. <https://doi.org/10.3109/19401736.2015.1060430>

Huang J, Xiang Y, Wu T, Zhang Y-X, Zhang Z-L, Wang B-Z, Lan X-Y, Huang Y-P, Jiang H-J, Jiang W-S (2024) Description of a new species of the Asian newt genus *Tylototriton* (Amphibia, Urodela, Salamandridae) from Hunan Province, China. Herpetozoa (Wien) 37: 327–338. <https://doi.org/10.3897/herpetozoa.37.e135222>

Jiang Y, Li Z, Liu J, Li Y, Ni Q, Yao Y, Xu H, Li Y, Zhang M (2015a) The complete mitochondrial genome sequence of *Tylototriton taliangensis* (Amphibia: Caudata). Mitochondrial DNA. Part A, DNA Mapping, Sequencing, and Analysis 27(4): 2639–2640. <https://doi.org/10.3109/19401736.2015.1041132>

Jiang Y, Yang M, Han F, Li Y, Ni Q, Yao Y, Xu H, Li Y, Zhang M (2015b) The complete mitochondrial genome sequence of red knobby newt *Tylototriton shanjing* (Amphibia: Caudata). Mitochondrial DNA. Part A, DNA Mapping, Sequencing, and Analysis 27(4): 2773–2774. <https://doi.org/10.3109/19401736.2015.1053059>

Jiang Y, Wei Z, Han F, Ni Q, Yao Y, Xu H, Li Y, Rao D, Zhang M (2017) The complete mitogenome sequence of *Tylototriton ziegleri* (Amphibia: Caudata). Conservation Genetics Resources 9(3): 503–506. <https://doi.org/10.1007/s12686-017-0710-8>

Khatiwada JR, Wang B, Ghimire S, Paudel S, Jiang J (2015) A new species of the genus Tylototriton (Amphibia: Urodela: Salamandridae) from eastern Himalaya. Asian Herpetological Research 6: 245–256. <https://doi.org/10.16373/j.cnki.ahr.170013>

Le D, Nguyen T, Nishikawa K, Lan Hung Son N, Nguyen H, Pham A, Matsui M, Bernardes M, Nguyen T (2015) A new species of *Tylototriton* Anderson, 1871 (Amphibia: Salamandridae) from northern Indochina. Current Herpetology 34(1): 38–50. <https://doi.org/10.5358/hsj.34.38>

Li X, Jiang Y, Li Y, Ni Q, Yao Y, Xu H, Zhang M (2015) Complete mitochondrial genome sequence of red-tailed knobby newt (*Tylototriton kweichowensis*). Mitochondrial DNA. Part A, DNA Mapping, Sequencing, and Analysis 27(6): 4336–4337. <https://doi.org/10.3109/19401736.2015.1089489>

Li S, Wei G, Cheng Y, Zhang B, Wang B (2020) Description of a new species of the Asian newt genus *Tylototriton* sensu lato (Amphibia: Urodela: Salamandridae) from southwest China. Asian Herpetological Research 11(4): 282–296. https://doi.org/10.16373%2Fj.cnki.ahr.200026

Li S-Z, Liu J, Shi S-C, Wei G, Wang B (2022) Description of a new species of the newt genus *Tylototriton* sensu lato (Amphibia: Urodela: Salamandridae) from southwestern China. Zootaxa 5128(2): 248–268. <https://doi.org/10.11646/zootaxa.5128.2.5>

Liu S, Hou M, Rao D (2022) Confirmation of *Tylototriton ziegleri* Nishikawa, Matsui &amp; Nguyen, 2013 in China, with discussion on the relationship between *T. verrucosus* Anderson, 1871 and *T. panwaensis* Grismer, Wood, Quah, Thura, Espinoza & Murdoch, 2019 (Caudata, Salamandridae). Biodiversity Data Journal 10: e82707. <https://doi.org/10.3897/BDJ.10.e82707>

Lyu Z-T, Wang J, Zeng Z-C, Zhou J-J, Qi S, Wan H, Li Y-Y, Wang Y-Y (2021) A new species of the genus *Tylototriton* (Caudata, Salamandridae) from Guangdong, southern China, with discussion on the subgenera and species groups within the genus. Vertebrate Zoology 71: 697–710. <https://doi.org/10.3897/vz.71.e73563>

Nishikawa K, Matsui M, Nguyen TT (2013a) A new species of *Tylototriton* from northern Vietnam (Amphibia: Urodela: Salamandridae). Current Herpetology 32(1): 34–49. <https://doi.org/10.5358/hsj.32.34>

Nishikawa K, Khonsue W, Pomchote P, Matsui M (2013b) Two new species of *Tylototriton* from Thailand (Amphibia: Urodela: Salamandridae). Zootaxa 3737(3): 261–279. <https://doi.org/10.11646/zootaxa.3737.3.5>

Nishikawa K, Rao D-Q, Matsui M, Eto K (2015) Taxonomic Relationship between *Tylototriton daweishanensis* Zhao, Rao, Liu, Li and Yuan, 2012 and *T. yangi* Hou, Li and Lu, 2012 (Amphibia: Urodela: Salamandridae). Current Herpetology 34(1): 67–74. <https://doi.org/10.5358/hsj.34.67>

Phimmachak S, Aowphol A, Stuart BL (2015) Morphological and molecular variation in *Tylototriton* (Caudata: Salamandridae) in Laos, with description of a new species. Zootaxa 4006(2): 285–310. <https://doi.org/10.11646/zootaxa.4006.2.3>

Phung TM, Pham CT, Nguyen TQ, Ninh HT, Nguyen HQ, Bernardes M, Le ST, Ziegler T, Nguyen TT (2023) Southbound—The southernmost record of *Tylototriton* (Amphibia, Caudata, Salamandridae) from the Central Highlands of Vietnam represents a new species. ZooKeys 1168: 193–218. <https://doi.org/10.3897/zookeys.1168.96091>

Pomchote P, Khonsue W, Sapewisut P, Eto K, Nishikawa K (2020) Discovering a population of *Tylototriton verrucosus* (Caudata: Salamandridae) from Thailand: implications for conservation. Tropical Natural History 20(1): 1–15. <https://doi.org/10.58837/tnh.20.1.210006>

Pomchote P, Peerachidacho P, Hernandez A, Sapewisut P, Khonsue W, Thammachoti P, Nishikawa K (2021) A new species of the genus *Tylototriton* (Urodela, Salamandridae) from western Thailand. ZooKeys 1072: 83–105. <https://doi.org/10.3897/zookeys.1072.75320>

Pomchote P, Peerachidacho P, Khonsue W, Sapewisut P, Hernandez A, Phalaraksh C, Siriput P, Nishikawa K (2024) The seventh species of the newt genus *Tylototriton* in Thailand: A new species (Urodela, Salamandridae) from Tak Province, northwestern Thailand. ZooKeys 1215: 185–208. <https://doi.org/10.3897/zookeys.1215.116624>

Poyarkov N, Nguyen T, Arkhipov D (2021) A new species of the genus *Tylototriton* (Amphibia, Caudata, Salamandridae) from central Vietnam. Taprobanica, the Journal of Asian Biodiversity 10(1): 4–22. <https://doi.org/10.47605/tapro.v10i1.244>

Poyarkov NA, Nguyen TV, Le SX, Arkhipov DV, Gorin VA, Hernandez A, Dufresnes C (2024) Multiple lines of evidence for a new cryptic species of *Tylototriton* (Amphibia, Salamandridae) from northern Vietnam. Alytes 41(1–4): 56–98. https://www.biotaxa.org/Alytes/article/view/86220

Stuart B, Phimmachak S, Sivongxay N, Robichaud W (2010) A new species in the *Tylototriton asperrimus* group (Caudata: Salamandridae) from central Laos. Zootaxa 2650(1): 19–32. <https://doi.org/10.11646/zootaxa.2650.1.2>

Sun X, Ding M, Xiao N, Li K, Pan T, Zhou J, Zhang B (2016) The complete mitochondrial genome of *Tylototriton kweichowensis* and implications for *Tylototriton* taxonomy. Mitochondrial DNA. Part B, Resources 1(1): 647–648. <https://doi.org/10.1080/23802359.2016.1180558>

Wang B, Nishikawa K, Matsui M, Nguyen T, Xie F, Li C, Khatiwada J, Zhang B, Gong D, Mo Y, Wei G, Chen X, Shen Y, Yang D, Xiong R, Jiang J (2018) Phylogenetic surveys on the newt genus *Tylototriton* sensu lato (Salamandridae, Caudata) reveal cryptic diversity and novel diversification promoted by historical climatic shifts. PeerJ 6: e4384. <https://doi.org/10.7717/peerj.4384>

Wang J-X, Lan X-Y, Luo Q-H, Gu Z-R, Zhou Q, Zhang M-Y, Zhang Y-X, Jiang W-S (2022) Characterization, comparison of two new mitogenomes of crocodile newts *Tylototriton* (Caudata: Salamandridae), and phylogenetic implications. Genes 13(1878): 1–13. <https://doi.org/10.3390/genes13101878>

Weisrock DW, Papenfuss TJ, Macey JR, Litvinchuk SN, Polymeni R, Ugurtas IH, Zhao E, Jowkar H, Larson A (2006) A molecular assessment of phylogenetic relationships and lineage accumulation rates within the family Salamandridae (Amphibia, Caudata). Molecular Phylogenetics and Evolution 41(2): 368–383. <https://doi.org/10.1016/j.ympev.2006.05.008>

Yang D, Jiang J, Shen Y, Fei D (2014) A new species of the genus *Tylototriton* (Urodela: Salamandridae) from northeastern Hunan Province, China. Asian Herpetological Research 5(1): 1–11. <https://doi.org/10.3724/SP.J.1245.2014.00001>

Yuan Z-Y, Jiang K, Lu S-Q, Yang J-X, Nguyen QT, Nguyen TT, Jin J-Q, Che J (2011) A phylogeny of the *Tylototriton asperrimus* group (Caudata: Salamandridae) based on a mitochondrial study: suggestions for a taxonomic revision. Dong Wu Xue Yan Jiu = Zoological Research 32(6): 577–584. https://doi.org/10.3724/sp.j.1141.2011.06577

Zaw T, Yin S, Chit MT, Hmwe KS (2020) Occurrence of *Tylototriton* species in Kachin State with emphasis on their morphology and molecular analysis. University of Mandalay Research Journal 11: 59–72.

Zhang P, Papenfuss TJ, Wake MH, Qu L, Wake DB (2008) Phylogeny and biogeography of the family Salamandridae (Amphibia: Caudata) inferred from complete mitochondrial genomes. Molecular Phylogenetics and Evolution 49(2): 586–597. <https://doi.org/10.1016/j.ympev.2008.08.020>

Zhang M, Han F, Ye J, Ni Q, Li Y, Yao Y, Xu H (2018a) The entire mitochondrial genome of Vietnam Warty Newt *Paramesotriton deloustali* (Salamandridae: Paramesotriton) with a new distribution record from China. Conservation Genetics Resources 10(3): 551–554. <https://doi.org/10.1007/s12686-017-0804-3>

Zhang M, Jiang Y, Han F, Ni Q, Li Y, Yao Y, Xu H (2018b) The complete mitochondrial genome of Myanmar warty newt *Tylototriton shanorum* (Salamandridae: *Tylototriton*). Conservation Genetics Resources 10(3): 321–323. <https://doi.org/10.1007/s12686-017-0813-2>
